# Resilience against resistance: the future of antimicrobial resistance in Mexico

**DOI:** 10.1099/jmm.0.002215

**Published:** 2026-10-13

**Authors:** Bonnie Evans, Charlotte Holtum, Santiago Castillo-Ramírez, Tania Rivera-Hernández, Roberto Velez, Mariana Castillo, Pablo Velasco, Renata Martínez, Jorge Garduño

**Affiliations:** 1 Microbiology Society, London, UK; 2 Centro de Ciencias Genómicas, Universidad Nacional Autónoma de México, Cuernavaca, México; 3 Unidad de Investigación Médica en Inmunoquímica, Unidad Médica de Alta Especialidad, Centro Médico Nacional Siglo XXI, Instituto Mexicano del Seguro Social, Ciudad de México, México; 4 British Embassy Mexico, Ciudad de México, México

**Keywords:** antimicrobial resistance, Mexico, One Health, policy

## Abstract

Antimicrobial resistance (AMR) is a serious and growing threat in Mexico. To support cross-sector dialogue and policy development around AMR, the Science and Technology Network, the Department for Business and Trade and the Microbiology Society convened a one-day workshop at the British Embassy in Mexico City. This commentary synthesises the key themes from the workshop discussions and presents the desired outcomes, challenges and policy interventions identified by participants.

## Introduction

Antimicrobial resistance (AMR) is an urgent and rapidly escalating global threat to the health of humans, animals and the environment. In 2021, Mexico ranked 104 out of 204 countries for the age-standardized AMR mortality rate [1]. AMR was associated with an estimated 66,500 deaths in Mexico – nearly twice the number of deaths from chronic respiratory diseases [1]. Mexico is part of the Latin America and the Caribbean super-region, which is projected to have the highest all-age mortality rate from AMR globally by 2050 [2].

Since its launch in Mexico in 2023, the Science and Technology Network (STN) at the British Embassy in Mexico City has supported initiatives to raise awareness of AMR, aligning with the Network’s thematic priority of global health. The STN, part of the Foreign, Commonwealth and Development Office and the Department for Science, Innovation and Technology, operates across 65 locations, identifying opportunities, fostering partnerships and driving collaboration to deliver mutual benefits for the UK and host countries. In parallel, the Department for Business and Trade’s Health and Life Sciences Sector Team at the British Embassy in Mexico City has been working with British health and life sciences sector companies in Mexico to engage with AMR as part of their corporate social responsibility.

In October 2023, the Microbiology Society launched ‘Knocking Out AMR’ – an organization-wide project to promote meaningful action on AMR both in the UK and globally. To kick-start this project, the Society hosted a series of workshops in the UK focusing on priority solution areas: diagnostics, surveillance, therapeutics and vaccines [3, 4]. The workshops used a complex systems mapping approach to support invited experts in mapping the opportunities, challenges and potential interventions within their solution area to tackle AMR effectively.

Inspired by these workshops, the STN partnered with the Microbiology Society and the Health and Life Sciences Sector Team to bring the Society’s systems-mapping approach to Mexico. The resulting workshop, ‘Resilience against Resistance: The Future of Antimicrobial Resistance in Mexico’, was held at the British Embassy in Mexico City in November 2025 and marked the Society’s first AMR workshop outside the UK.

This workshop aimed to map the AMR landscape in Mexico, while fostering international dialogue, knowledge exchange and strategic planning around AMR challenges. It also sought to align with the Mexican Government’s national priorities of public health and scientific innovation to ensure action. The workshop was designed to promote a One Health approach to tackling AMR, recognizing the interconnectedness of human, animal and environmental health and the need for coordinated action across these domains [5].

This article reports on the workshop and presents a synthesis of the desired outcomes, challenges and policy interventions identified by participants.

### Workshop participants

Participants were selected for the workshop if they had recognized expertise in AMR and a professional affiliation in Mexico. To support representation across stakeholder groups, participants were also categorized by sector (academia, healthcare, policy or industry), One Health domain (human, animal or environment), AMR solution area (diagnostics, surveillance, therapeutics or vaccines) and micro-organism of interest (bacteria, viruses or fungi).

Workshop participants were identified by the Microbiology Society through its membership and publishing networks, recommendations from the workshop co-chairs and two additional contacts from Mexico, and desk research. The STN and the Health and Life Sciences Sector Team also facilitated introductions through their professional networks to relevant stakeholders from Mexican government institutions. Invitations were issued by the British Embassy in Mexico City.

A total of 70 experts participated in the workshop, representing academia (35% of participants), healthcare (32%), policy (27%) and industry (7%). More than 75% of participants had expertise in AMR within human health, with the remaining participants representing animal, environment or broader One Health perspectives.

### Workshop structure

For the morning session, participants were split into breakout groups of mixed expertise to produce systems maps of AMR in Mexico. Participants first envisioned what the AMR landscape would look like in Mexico in a perfect world. They then identified challenges that prevented progress towards this perfect-world scenario and proposed policy interventions that could overcome these challenges. Facilitators mapped participants’ contributions during the discussions, adding and linking the perfect-world scenarios, challenges and policy interventions as they were identified by the group.

In the afternoon session, participants were divided into groups according to their areas of expertise: vaccines, diagnostics and therapeutics, and surveillance and the environment. Participants were asked to consider how solutions to AMR could be integrated into the Plan Mexico strategy, which sets out national development goals to be achieved by 2030 [6], specifically:

- Increase the use of domestically manufactured content in pharmaceutical and other industrial products by 15%.
- Vaccines made in Mexico – drive the complete development of pharmaceutical manufacturing processes and local packaging with an emphasis on advanced biotechnology.
- Environmental sustainability – promote investments with environmental, social and governance practices: water reuse, investment in clean energy with backup, solid waste management systems and community impact actions.
- Provide 30% of small and medium-sized enterprises with access to financing.

Each breakout group activity was followed by a presentation of the group’s findings to the full workshop assembly, which then led into a wider discussion. Note-takers documented discussions in each breakout group and plenary session.

### Synthesis of workshop outputs

Following the workshop, hard copies of the systems maps were digitized and translated from Spanish to English by the British Embassy in Mexico City. Artificial Intelligence (AI)-assisted translation (Google Translate) was used to translate discussion notes into English.

Author B.E. reviewed the translated outputs and identified recurrent themes across the breakout groups. Although discussions covered a wide range of topics, common themes included surveillance, antimicrobial stewardship across the pipeline (design, access, use and disposal), diagnostics, infection prevention and control (IPC) (with an emphasis on vaccination), policy engagement and education. B.E. then identified the most frequently discussed perfect-world scenarios, challenges and policy interventions within these thematic areas, which are summarized below.

To visualize the key workshop findings, B.E. developed a systems map of perfect-world scenarios, challenges and policy interventions, and the connections between them (Fig. 1). The final network of connections reflects both links identified by participants during the workshop and author interpretation undertaken during the synthesis process.

**Fig. 1.**
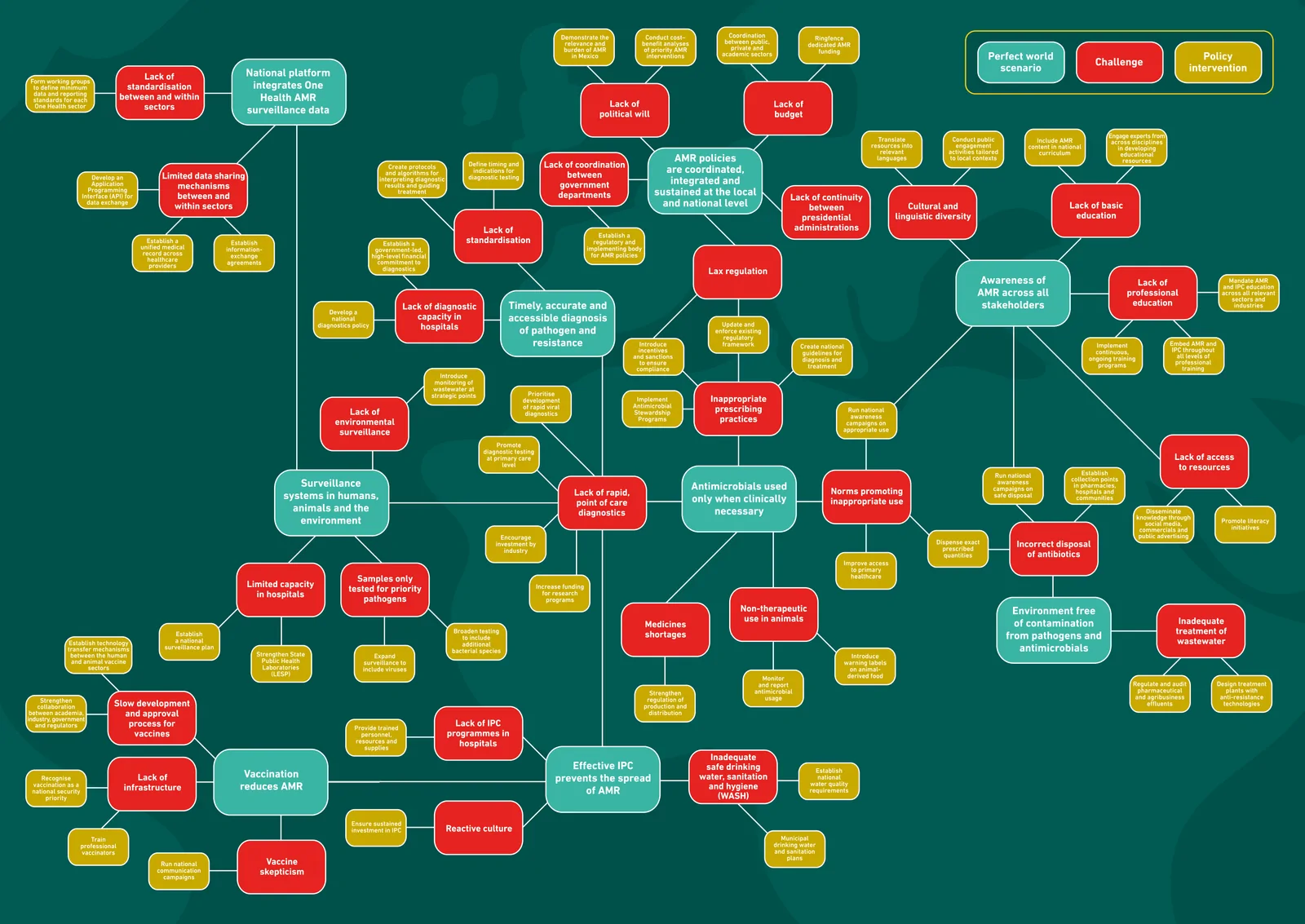
Summary systems map of AMR in Mexico. Teal boxes indicate aspects of a perfect-world scenario in which AMR is effectively managed in Mexico. Red boxes indicate current challenges that prevent this scenario from being achieved. Mustard yellow boxes indicate policy interventions that could help address challenges and move towards the perfect world scenario. The lines between boxes show the connections between these elements.

Authors C.H., S.C.-R., T.R.-H. and R.V. reviewed the synthesis of the workshop outputs and confirmed that it accurately reflected the perspectives expressed by participants.

## Workshop summary

### Surveillance

Participants emphasized the need for a comprehensive One Health AMR surveillance system so that the AMR situation in Mexico can be fully understood and addressed. Currently, surveillance efforts remain siloed between and within One Health sectors. Mexico lacks a nationally coordinated and standardized AMR surveillance system for human health. Instead, several international, governmental and non-governmental networks and reference centres operate independently. Participants perceived that this fragmentation is compounded by the complexity of Mexico’s healthcare system, where each healthcare provider operates its own closed network of hospitals, doctors and pharmacies. While Mexico has a well-established national AMR surveillance system in food animals, this system lacks communication or coordination with human health. Furthermore, there is no formal monitoring of resistance or antimicrobials in the environment, leaving a significant gap in present AMR surveillance.

#### In a perfect world

- Resistance data across the human, animal, agrifood and environmental sectors are fully integrated within a single, centralized platform.
- Real-time reporting of resistance data supports early detection of trends, outbreaks and zoonoses.
- Reporting occurs at national, regional and local levels.
- Clinicians have access to resistance data that directly informs prescribing practices and IPC actions.
- Surveillance systems actively monitor environmental contaminants, including antimicrobial residues and metals that drive cross-resistance to antimicrobials.

#### Challenges

- A fragmented healthcare system with limited data-sharing mechanisms.
- Surveillance is not implemented in all hospitals due to deficits in diagnostic laboratories and tools, human and financial resources.
- Lack of point-of-care, rapid diagnostics limits community, primary-level data collection.
- Lack of standardization of data due to differences in collection, testing and analysis of samples across the One Health domains.

#### Policy interventions

- Develop, evaluate and finance a national surveillance plan.
- Create an independent entity that is responsible for the integrated data platform and reporting.
- Establish collaboration and information exchange agreements between institutions.
- Strengthen local epidemiology and the State Public Health Laboratories.
- Mandate reporting into the national data platform.
- Create a unified, electronic medical record.

### Antimicrobial stewardship across the pipeline

Participants consistently expressed concerns about the inappropriate prescribing and use of antimicrobials, while also emphasizing the importance of universal access to appropriate treatment in a perfect-world scenario. They noted that the livestock sector is the largest consumer of antibiotics in Mexico, raising concerns about agricultural practices and their contribution to AMR in the environment. Poor waste management practices and improper disposal of antibiotics were also identified as key issues that lead to environmental contamination with antimicrobials. Participants highlighted the need for stronger regulation and improved education for both the public and professionals to ensure effective stewardship across the antimicrobial pipeline.

#### In a perfect world

- Antimicrobials are prescribed only when clinically necessary.
- Treatment regimens, including drug choice, dose and timing, are personalized and optimized for each patient.
- Antimicrobial use in animals is restricted to justified therapeutic purposes.
- Antimicrobials are disposed of safely and have short half-lives.
- Novel, narrow-spectrum antimicrobials and alternative treatment options, such as immunotherapies and bacteriophage therapies, are available for infections.

#### Challenges

- Prescribing decisions are influenced by factors other than clinical need, including:
  - Patient expectations, such as the belief that care is inadequate if antimicrobials are not prescribed.
  - Supply issues with antibiotics, which can lead to the use of less appropriate or broader-spectrum alternatives.
  - Inadequate availability of other medicines, for example analgesics or antipyretics needed to manage symptoms.
  - Limited time available to see and treat patients.
  - Financial incentives – many private pharmacies operate on-site or co-located medical consulting rooms to increase medicines sales through pharmacy dispensing. These services are popular due to their accessibility, low or no consultation costs and delivery by licensed medical practitioners.
- Diagnostic tools are not available at point-of-care to guide antimicrobial prescribing.
- Cephalosporins, many of which are broad-spectrum agents, are overprescribed, with limited use of other antibiotic classes.
- Self-medication with antibiotics leftover from previous treatments or obtained from family members is common practice.
- Antimicrobials are discarded through normal household waste routes.
- Antibiotics are discouraged but not fully banned for prophylactic use or growth promotion, including via medicated feed, in the livestock industry.

#### Policy interventions

- Implement and monitor Antimicrobial Stewardship Programmes in all hospitals, with regular audits to evaluate compliance.
- Educate the population and prescribers on the appropriate use and disposal of antimicrobials through a national communication campaign.
- Strictly regulate the production, distribution and sale of antimicrobials.
- Mandate monitoring and reporting of antibiotic sales from all prescribers, with sanctions for non-compliance.
- Dispense antimicrobials in the precise amount needed to complete the treatment regimen.
- Require wastewater, such as from hospitals, farms and pharmaceutical factories, to be treated appropriately to remove antimicrobial residues and resistant micro-organisms before discharge into the environment.
- Repurpose and expand the National Service for Agrifood Health, Safety and Quality’s antibiotic traceability system to monitor antimicrobial consumption and usage.
- Require clear labelling of food products where antimicrobials were used during production.

### Diagnostics

Participants frequently used the same three words to describe what diagnostic tests for infectious diseases would look like in a perfect world: timely, accessible and accurate. While some diagnostic testing is available at the tertiary level of care in Mexico, there was broad agreement that there needed to be a shift towards primary level or point-of-care diagnostics to reduce unnecessary antibiotic consumption. As well as antimicrobial stewardship, diagnostics was viewed as essential for AMR surveillance and IPC. Opinions differed on the primary bottleneck within diagnostic laboratories. Some highlighted shortages of supplies; others pointed to insufficient numbers of trained personnel or inadequate infrastructure. However, participants noted that these issues ultimately stem from budget constraints and the perception that diagnostics are secondary to hospitals’ more immediate needs. As a result, a high-level financial commitment is required to expand diagnostic capacity across all levels of healthcare if AMR is to be effectively addressed.

#### In a perfect world

- Antimicrobials are only prescribed after identifying the causative pathogen and assessing its resistance profile.
- Rapid diagnostic tests are available at point-of-care and are accessible to the population, bringing the ‘laboratory to the home’.
- Primary level healthcare has an easy route to sending samples to diagnostic laboratories.
- Hospitals have the budget, infrastructure and workforce for diagnostic laboratories, and diagnostic testing is embedded in hospital protocols for infectious diseases.
- Diagnostic results are accurate and provided in time to support appropriate treatment and prognosis.
- Diagnostic results from every level of the healthcare system are systematically incorporated into AMR and epidemiological surveillance.

#### Challenges

- Diagnostic tests are not routinely requested or available, and infections are treated empirically with antimicrobials.
- Turnaround for antimicrobial susceptibility testing is slow, for example antibiograms can take 24 to 72 h, which means they cannot guide initial treatment decisions.
- There is a scarcity of supplies needed to run diagnostic tests and quality control, including reagents, consumables and reference materials.
- Diagnostics are not fully implemented across hospitals, many of which lack laboratories capable of performing even basic microbiology techniques.
- Hospitals lack essential infrastructure, such as isolation rooms and automated systems, to deliver reliable and timely diagnosis.
- Shortage of skills, training and jobs to support diagnostic services.

#### Policy interventions

- Perform cost-benefit analysis to convince decision makers to invest in diagnostics.
- Prioritize the development of rapid tests for viruses, preventing improper use of antibiotics for viral infections.
- Identify the most needed diagnostic tools using healthcare worker feedback and surveillance data.
- Develop clear hospital protocols for when diagnostic testing should be used.

### Infection prevention and control (IPC)

Participants frequently commented that Mexico has a ‘reactive rather than preventative culture’. Funding for IPC is typically mobilized in response to outbreaks, rather than through sustained investment in prevention efforts that could help reduce AMR. Participants recognized that vaccines are a critical tool for preventing the development of AMR, reducing the spread of AMR-relevant pathogens and minimizing unnecessary antibiotic use.

#### In a perfect world

- Universal access to safe drinking water, sanitation and hygiene and vaccination.
- A national IPC programme is established, fully operational and continuously improved.
- Early detection of public health risks through integrated diagnostics and surveillance data, enabling timely preventative measures and targeted vaccination.
- Vaccines are developed and/or improved for AMR-relevant pathogens endemic to Mexico.
- Vaccine sovereignty – Mexico has the national capacity to design, produce and distribute vaccines.

#### Challenges

- Lack of access to a safe, continuous supply of water and to secure sanitation systems for all households.
- Insufficient infrastructure supplies and human resources to support effective IPC and vaccination programmes in hospitals and communities.
- Scepticism about the necessity or safety of vaccination amongst the population.
- Deficits in the cold chain for distributing and storing vaccines.
- Slow and bureaucratic approval of vaccines by the Federal Commission for the Protection against Sanitary Risks.
- Time and financial commitment needed for vaccine development is greater than the length of political terms, meaning projects can be dropped.
- Vaccines developed and made in Mexico must compete with those made by large, multinational companies.

#### Policy interventions

- Develop and implement municipal drinking water and sanitation plans.
- Expand professional education on prevention, embedding IPC and AMR content at all training levels.
- Run national education campaigns to encourage vaccine uptake and tackle misinformation.
- Strengthen collaboration between academia, industry, government and regulators to ensure that regulation is fit for purpose and allows for vaccine innovation.
- Guarantee a market for domestically produced vaccines by establishing public–private partnerships.
- Elevate vaccine governance to the highest levels of decision-making, recognizing that vaccine production and distribution are matters of national security.
- Strengthen collaboration between human and animal vaccine producers to accelerate technology adoption and share lessons learnt.

## Discussion

The workshop brought together 70 experts from academia, healthcare, policy and industry to discuss AMR in Mexico. Two key outcomes emerged from the workshop. First, the event facilitated cross-sectoral learning and relationship-building among individuals and organizations that may not otherwise interact regularly. Second, participants identified priorities, challenges and potential areas for action. Key cross-cutting challenges identified included fragmented data systems, limited resources and lack of coordination between and within sectors. Participants highlighted the need for an integrated, national One Health surveillance system, improved antimicrobial stewardship across human, animal and environmental sectors, access to rapid, point-of-care diagnostics and sustained investment in preventative measures.

However, the workshop findings should be considered in light of several limitations. They reflect the perspectives and expertise of the workshop participants and authors and, although efforts were made to engage a broad range of stakeholders, may not represent the full diversity of views held across Mexico. Despite the workshop being designed to promote a One Health approach to tackling AMR, participants were at times hesitant to take a more cohesive view across human, animal and environmental domains. This may partly reflect the participant composition, as most attendees had expertise in human health. As a 1-day workshop with a broad remit to map the AMR landscape in Mexico, the findings also reflect the priorities raised during discussions rather than a systematic assessment of national AMR needs. Finally, given the breadth of the discussions and the diversity of stakeholders involved, the policy interventions identified were relatively high-level and should be considered as a starting point for further exploration.

Future work should refine the proposed policy interventions to ensure they are tailored to the Mexican context and practical to implement. More focused stakeholder workshops centred on the four themes identified, namely surveillance, antimicrobial stewardship, diagnostics and IPC, could help assess the feasibility, implementation requirements and timelines of policy interventions.

Strengthening engagement with Mexican government departments and agencies across the human health, animal health and environmental sectors may also support implementation of these interventions and promote a more integrated One Health approach to AMR policy. In parallel, expanding similar multi-sectoral dialogues to other Latin American countries, and more broadly across low- and middle-income countries, could strengthen regional and global efforts to address AMR. This work is already being advanced by the STN in Mexico through collaboration and knowledge exchange with counterparts in Brazil, Chile and Argentina.

## Abbreviations

AI: artificial intelligence
AMR: antimicrobial resistance
IPC: infection prevention and control
STN: Science and Technology Network
